# Effects of narrowband ultraviolet B exposure on serum 25-hydroxyvitamin D concentrations: A pilot study

**DOI:** 10.1097/MD.0000000000029937

**Published:** 2022-08-19

**Authors:** Seok-Hoon Lee, Nam-Seok Joo

**Affiliations:** Department of Family Practice and Community Health, Ajou University School of Medicine, Suwon, Republic of Korea.

**Keywords:** 25-hydroxyvitamin D, light-emitting diode, narrowband ultraviolet B, vitamin D, ultraviolet B

## Abstract

It is known that ultraviolet B exposure increases serum 25-hydroxyvitamin D(25(OH)D) concentrations. However, little is known about the influence of narrowband ultraviolet B exposure from a light-emitting diode (NBUVB-LED) on serum 25(OH)D levels. Thus, the purpose of this study was to investigate the effect of NBUVB-LED exposure on serum 25(OH)D concentrations.

Two healthy adults were enrolled in this pilot study. Their skin was exposed to ultraviolet B light (60 mJ/cm^2^) 3 times a week for 4 weeks in the first intervention and every day for 4 weeks in the second intervention. Serum levels of 25(OH)D were measured every 2 weeks.

Serum 25(OH)D levels were decreased in both subjects at the end of the first intervention (32.1 → 21.4 ng/mL, 33.9 → 21.4 ng/mL, respectively), whereas serum 25(OH)D levels were increased in the 2 weeks of the second intervention (29.5 and 28.0 ng/mL, respectively). At the end of the second intervention, the 25(OH)D concentrations were 19.0 and 20.4 ng/mL, respectively.

NBUVB-LED exposure might increase serum 25(OH)D concentrations. Future studies should expand the number of participants and adjust for confounding factors.

## 1. Introduction

Vitamin D deficiency is a global issue and a recent health concern of Koreans.^[1]^ Vitamin D not only affects bones and muscles but also affects chronic conditions, such as type 2 diabetes mellitus, hypertension, autoimmune diseases, infectious diseases, and various types of cancer.^[2–4]^ It is mainly acquired by dermal synthesis in response to ultraviolet B (UVB) sun radiation. The dietary sources of vitamin D are limited.^[5]^

In summer in Korea (36°N), about 30 minutes of exposure to the forearms and face at least 3 times a week during midday is considered to be sufficient for maintaining serum vitamin D concentrations.^[6]^ However, drastic changes in modern environments, including indoor lifestyles and the novel SARS-CoV-2 outbreak, have made vitamin D deficiencies one of the most prevalent health problems.^[7]^ Other atmospheric conditions, such as ozone and suspended particles, can also decrease UVB radiation.^[8]^ In this situation, vitamin D supplementation is recommended to maintain adequate serum 25-hydroxyvitamin D (25(OH)D) concentrations. However, daily supplementation is not easy for some populations. Young healthy people, old people, and patients who have medical conditions, such as bowel disease, fat malabsorption, and obesity, are at risk of vitamin D deficiency.^[9,10]^ Moreover, the recommended optimal vitamin D intake has increased.^[11,12]^ Thus, it may be wise to find an additional way to maintain adequate serum vitamin D concentrations.

UVB from the sun or artificial lights can stimulate the photosynthesis of vitamin D in human skin.^[13,14]^ This wavelength range is the only part of the ultraviolet radiation spectrum that produces vitamin D.^[15]^ Previous studies showed that serum 25(OH)D concentrations were significantly increased in healthy subjects with UVB exposure.^[16,17]^ Therefore, artificial UVB exposure might be an additional method to increase serum 25(OH)D levels.

In clinical practice, UVB exposure is considered an easy and cost-effective means of increasing 25(OH)D levels. The action spectrum for the conversion of 7-dehydrocholesterol to 25(OH)D is thought to be within the specific UVB wavelengths of 290–315 nm.^[18]^ Compared to broad-band ultraviolet light, a narrowband ultraviolet lamp is therapeutically more efficient and better tolerated. It avoids harmful and unnecessary UVB radiation wavelengths while supplying 25(OH)D.^[19]^ However, little is known about the efficiency of narrowband UVB exposure from a light-emitting diode (NBUVB-LED) (295nm) to increase serum 25(OH)D levels. Thus, the purpose of this pilot study was to assess the effect of NBUVB-LED exposure on serum 25(OH)D concentrations.

## 2. Methods

### 2.1. Study subjects and design

This pilot study was conducted from April 2021 to September 2021. Two healthy young adults 20–60 years old with a body mass index of <25 kg/m^2^ were enrolled. The exclusion criteria were a prior diagnosis of osteoporosis (T-score < −2.5), chronic alcoholic liver disease, alcoholism, primary parathyroid disease, or metabolic bone disease; serum aspartate aminotransferase or alanine aminotransferase >120 mg/dL; serum creatinine >2.0 mg/dl; thyroid-stimulating hormone >10 μIU/mL or TSH < 0.15 μIU/mL; subjects who were taking medications such as antihypertensives, diabetes medications, steroids, diuretics, and vitamin D supplements within 3 months; a medical history of malignancy (including skin cancer); subjects who had any kind of dermatitis; subjects who had photo-allergies; and subjects whose skin color was too bright or dark. Two healthy men without medical history voluntarily participated in this study after providing written informed consent. After enrollment, the subjects were requested to visit the outpatient department every 2 weeks for a routine check for adverse events, as well as 25(OH)D measurements. This study was approved by the Institutional Review Board of Ajou University Hospital (AJIRB-MED-INT-20-590).

### 2.2. Intervention

**Figure 1.**
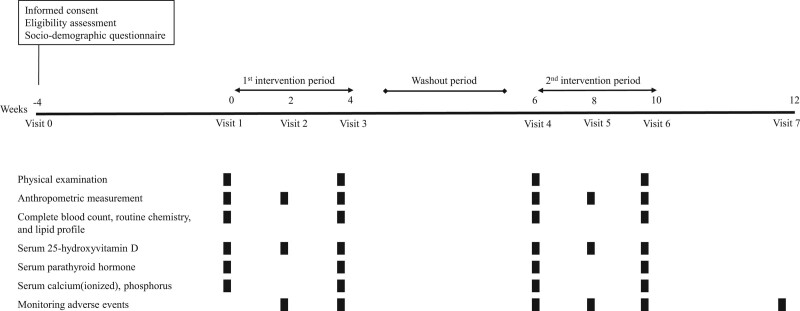
Diagram of the study schedule.

**Figure 2.**
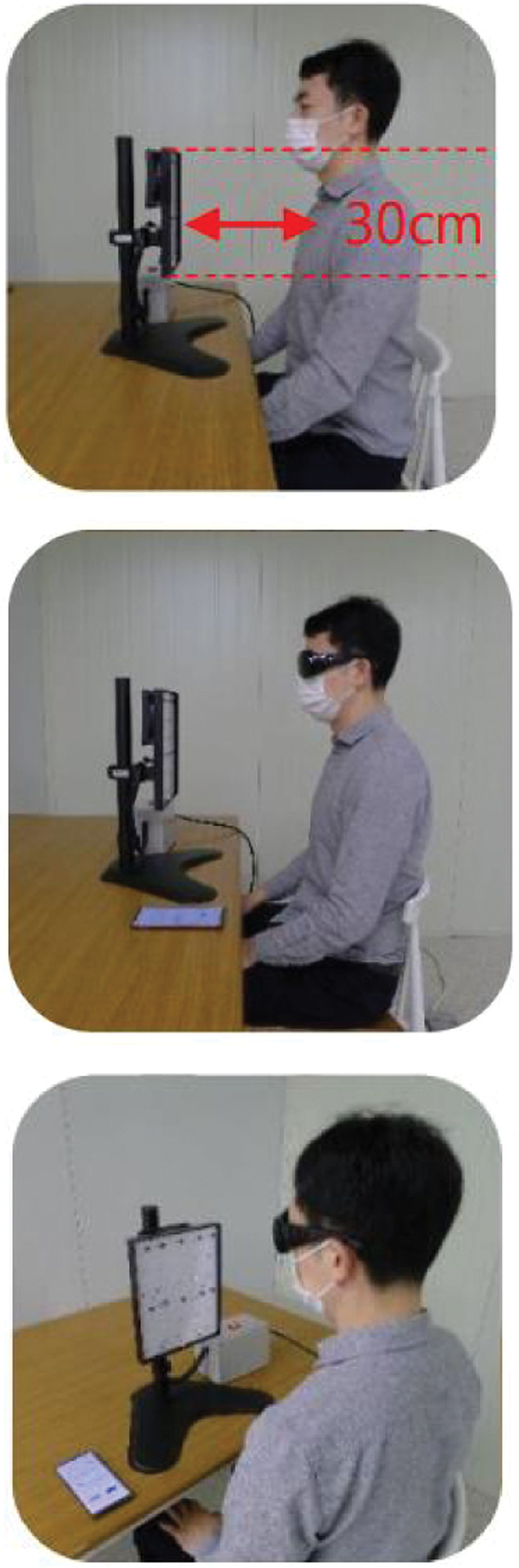
Photograph of actual NBUVB-LED radiation. During NBUVB-LED exposure, the subjects were requested to remove their shirts while wearing eye-protective goggles.

The intervention schedule is summarized in Figure 1. During the 2 months of the intervention, the subjects were requested to remove their shirts and sit in front of an NBUVB-LED while wearing protective goggles to prevent eye damage. The subjects were kept 30–50 cm from the light source panel, which was adjusted with a height controller to their upper body (Fig. 2). The exposed area included the face, the anterior aspects of both upper extremities, chest, and upper abdomen. They had about 30% skin exposure calculated from an adaptation of ‘‘the rule of nines,” representing adult body surface area.^[20]^

The subjects received 60 mJ/cm^2^ of UVB (a peak wavelength of 295 nm) light for 10 minutes in each treatment. Artificial ultraviolet light was provided by an NBUVB-LED from Seoul Viosys Co., Ltd. (Ansan, Republic of Korea). The lamp was controlled by a timer that turned off the light after 10 minutes. The dose was much lower than that used for ultraviolet light therapy for some kinds of dermatitis, which usually ranges from 300 to 500 mJ/cm^2^, and might cause erythema or rashes. Therefore, we assumed that the radiation dose would not be harmful to the skin. The participants were instructed not to use sunscreen when getting narrow-band UVB exposure. The dose was maintained throughout the study for each subject. During the 2 months of the intervention, the participants were exposed 3 times a week for 4 weeks in the first session and 7 times (every day) a week for 4 weeks in the second session. There was a washout period of 2 weeks between the 2 sessions. Serum 25(OH)D levels were measured every 2 weeks. Anthropometrical measurements and assessments of serum parathyroid hormone (PTH), ionized calcium, and phosphorus were conducted at visits 1, 3, 4, and 6. Two weeks after visit 6, phone calls were made to check whether any adverse events occurred. We also educated them to eat foods during the study as they usually had.

### 2.3. Body proportion measurements and laboratory assessments

Body proportion measurements, including body weight, muscle mass, fat mass, and fat % mass, were performed with a bio-impedance analysis device (InBody770, Biospace, Seoul, Korea). Blood samples for biochemical measurements were obtained from each subject. Serum concentrations of 25(OH)D, PTH, ionized calcium, and phosphorus were assessed. Serum 25(OH)D was assessed at every visit. Parathyroid hormone, serum calcium, and phosphorus were assessed at visits 1, 3, 4, and 6. The primary outcomes were differences in the serum levels of 25(OH)D in each subject at baseline, visit 3, and visit 6. Blood samples were collected year-round after an 8-hour fast. They were immediately processed, refrigerated, and transported in cold storage to the central testing institute (NeoDin Medical Institute, Seoul, South Korea), where they were analyzed within 24 hours. Serum 25(OH)D was measured with a radioimmunoassay kit (25(OH)D ^125^I RIA Kit; DiaSorin Inc., Stillwater, MN) using a gamma-counter (1470 Wizard; PerkinElmer, Turku, Finland). The inter-assay coefficients of variation were 13% and 10% at 31 nmol/L and 47 nmol/L, respectively, while the intraassay coefficients of variation were 5.1% and 4.9% at 30 nmol/L and 54 nmol/L, respectively. Serum 25(OH)D was measured in the same institute that conducted quality control assessments every other week throughout the analysis period to minimize analytical variation. Serum parathyroid hormone was analyzed using a chemiluminescence assay (DiaSorin). Ionized calcium was measured in whole blood with an ABL700 blood gas analyzer (Radiometer a/s, Denmark) using an E733 ion-selective electrode. The intraseries coefficient of variation was 0.66% at 1.6 mmol/L and the inter-series coefficient of variation was 1.24% at 1.6 mmol/L.

### 2.4. Statistical analysis

Descriptive statistics were used to present the data, mean, and standard deviation before and after the interventions in each subject. No formal statistical comparison testing was done.

## 3. Results

The baseline characteristics of the 2 subjects are presented in Table 1. The blood pressure of subject B was high. We assumed that it might be a white-coat effect since he had never had high blood pressure before. The baseline serum 25(OH)D levels were 32.1 and 33.9 ng/mL (80.3 and 84.8 nmol/L), respectively. Serum PTH levels were different between the 2 subjects (8.0 pg/mL and 27.0 pg/mL). Other baseline variables, such as liver enzymes, cholesterol, uric acid, calcium, and phosphate, were within the reference values.

**Table 1:** Baseline characteristics of subjects.

	Subject A	Subject B
	Visit 1	Visit 1
Age (yr)	25	25
Height (cm)	172.6	185.1
Weight (kg)	70.9	83.4
Body mass index (kg/m^2^)	23.8	24.3
Muscle mass (kg)	34.2	40.6
Body fat mass (kg)	10.8	12.8
Body fat percentage	15.2	15.4
Visceral fat mass (cm^2^)	40.7	54.1
Systolic blood pressure (mm Hg)	124	153
Diastolic blood pressure (mm Hg)	75	97
Smoker	No	Yes
Pack-year	–	2.25
Alcohol consumption	No	No
Co-morbidities	No	No
25-hydroxyvitamin D3 (ng/mL)	32.1	33.9
Parathyroid hormone	8.0	27.0
White Blood cell (10^3^/μL)	4.8	7.9
Hemoglobin (g/dL)	15.9	15.3
AST (mg/dL)	17	23
ALT (mg/dL)	15	23
ALP (mg/dL)	77	83
Total protein (mg/dL)	7.5	7.7
Albumin (mg/dL)	5.1	4.8
Total cholesterol (mg/dL)	152	219
Triglyceride (mg/dL)	95	94
HDL-Cholesterol (mg/dL)	57	60
LDL-Cholesterol(mg/dL)	76	140
Fasting glucose (mg/dL)	85	98
BUN (mg/dL)	13.0	18.6
Creatinine (mg/dL)	1.06	1.03
Uric acid (mg/dL)	5.3	5.2
Calcium (mg/dL)	9.8	9.8
Phosphorus (mg/dL)	3.2	3.1

AST = aspartate aminotransferase, ALT = alanine aminotransferase, ALP = alkaline phosphatase, HDL = high-density lipoprotein, LDL = low-density lipoprotein, BUN = blood urea nitrogen.

Table 2 shows changes in the anthropometric and biological markers of the 2 subjects. During the second intervention, subject A gained 3.4 kg of weight and 2.7 kg of muscle mass with decreases in body fat mass and visceral adipose tissue. The weight gain might have been due to sustained resistance exercise. Otherwise, there were no significant changes between the visits.

**Table 2:** Changes of anthropometric and biologic markers in subjects.

	Changes of anthropometric markers	
	Subject A: visit 1 → visit 6	Subject B: visit 1 → visit 6
Weight (kg)	70.9 → 74.3	83.4 → 83.9
Body mass index (kg/m^2^)	23.8 → 24.9	24.3 → 24.5
Muscle mass (kg)	34.2 → 36.9	40.6 → 40.6
Body fat mass (kg)	10.8 → 11.0	12.8 → 12.4
Body fat percentage	15.2 → 14.8	15.4 → 15.4
Visceral fat mass (cm^2^)	40.7 → 32.7	54.1 → 48.2

	Changes of biologic markers	
	Subject A: V1 → V3 → V6	Subject B: V1 → V3 → V6
White Blood cell (10^3^/μL)	4.8 → 5.1 → 5.3	7.9 → 6.5 → 7.4
Hemoglobin (g/dL)	15.9 → 15.9 → 15.5	15.3 → 15.7 → 15.2
AST (mg/dL)	17 → 32 → 20	23 → 40 → 29
ALT (mg/dL)	15 → 23 → 18	23 → 26 → 24
ALP (mg/dL)	77 → 76 → 65	83 → 84 → 80
Total protein (mg/dL)	7.5 → 7.3 → 7.4	7.7 → 7.2 →7.4
Albumin (mg/dL)	5.1 → 4.9 →4.8	4.8 → 4.5 →4.7
Total cholesterol (mg/dL)	152 → 155 → 145	219 → 209 → 205
Triglyceride (mg/dL)	95 → 88 → 50	94 → 167 → 85
HDL-Cholesterol (mg/dL)	57 → 58 → 56	60 → 47 → 49
LDL-Cholesterol (mg/dL)	76 → 79 → 79	140 → 129 → 139
Fasting glucose (mg/dL)	85 → 87 →89	98 → 82 → 93
BUN (mg/dL)	13.0 → 14.4 → 14.3	18.6 → 14.3 →15.0
Creatinine (mg/dL)	1.06 → 1.05 →1.02	1.03 → 1.13 → 1.08
Uric acid (mg/dL)	5.3 → 4.8 → 4.3	5.2 → 5.0 → 5.4
Calcium (mg/dL)	9.8 → 10.1 → 9.6	9.8 → 9.3 → 9.8
Phosphorus (mg/dL)	3.2 → 4.3 → 3.3	3.1 → 2.7 → 3.6

AST = aspartate aminotransferase; ALT = alanine aminotransferase; ALP = alkaline phosphatase; BUN = blood urea nitrogen; HDL = high-density lipoprotein; LDL = low-density lipoprotein; V1 = visit 1; V3 = visit 3; V6 = visit 6.

Table 3 summarizes the effects of radiating NBUVB-LED on 25(OH)D and PTH levels. Contrary to our expectation, the serum 25(OH)D levels of the subjects were decreased at the end of the first session, showing the same value of 21.4 ng/mL in both subjects. After the 2-week wash-out period, the serum 25(OH)D concentrations in subjects A and B were 26.5 and 25.5 ng/mL, respectively. After 2 weeks of daily exposure in the second session, the serum 25(OH)D levels in each subject showed subtle increases to 29.5 ng/mL and 28.0 ng/mL, respectively. However, subject A reported a tingling sensation on his skin at visit 6. We suspected that light exposure might have caused such a side effect as this symptom had never happened before. Thus, we recommended reducing the frequency of exposure in subject A to 3 times per week. The serum 25(OH)D levels of subject A were decreased after visit 7. The tingling symptom of subject A was improved. Except for this symptom, no other adverse effects were detected in this study. Finally, the last serum 25(OH)D concentrations were 19.0 ng/mL in subject A and 20.4 ng/mL in subject B.

**Table 3:** Changes in serum 25(OH)D and PTH concentrations after radiating with NBUVB-LED.

		1^st^ intervention				2^nd^ intervention		
		Visit 1	Visit 2	Visit 3		Visit 4	Visit 5	Visit 6
Subject A	25(OH)D (ng/mL)	**32.1**	22.8	**21.4**	Wash-out for 2 weeks	**26.5**	**29.5**	**19.0**
	PTH (pg/mL)	8.0		7.0		5.0		28.0
Subject B	25(OH)D (ng/mL)	**33.9**	24.1	**21.4**		**25.5**	**28.0**	**20.4**
	PTH (pg/mL)	27.0		38.0		9.0		46.0
Exposures		10 minutes, 3 times/week				10 minutes, 7 times/week		

NBUVB-LED = narrowband ultraviolet B from a light-emitting diode, 25(OH)D = 25-hydroxyvitamin D, PTH = parathyroid hormone.

## 4. Discussion

The results of this preliminary study can be summarized as follows. In healthy men, daily NBUVB-LED exposure to 30% of the body surface for 10 minutes tended to increase serum 25(OH)D concentrations, whereas 10 minutes of exposure 3 times a week did not. Most of the NBUVB-LED effects seemed to be associated with the frequency of exposure since increases in serum 25(OH)D concentrations occurred at the end of the first 2 weeks of the second intervention but returned to basal levels after reducing the frequency of exposure in subject A. This decline might have been due to decreased NBUVB-LED exposure since there was no significant increase in body fat mass.

The decreases in serum 25(OH)D concentrations at the end of the first and second intervention periods were unexpected. A previous study^[21]^ reported a similar phenomenon. Although phototherapy increases 25(OH)D levels, some patients did not show a significant increase. A few others even showed decreases in 25(OH)D concentrations. The reasons for such decreases are not yet fully understood. Several factors might contribute to this phenomenon. First, despite unknown mechanisms, 25(OH)D may act as negative feedback for 25-hydroxylase in the liver, which prompts the hydroxylation of vitamin D3 to 25(OH)D. The conversion of 25(OH)D to the 1,25-dihydroxyvitamin D metabolite could explain part of the decrease in 25(OH)D. Second, the subjects conducted the interventions themselves. The absence of a supervisor may have decreased compliance and introduced errors. Third, the decrease might have been caused by the severe acute respiratory syndrome coronavirus 2 pandemic since people spent less time outdoors with less sun exposure, consequently reducing the production of 25(OH)D. Fourth, sinusoidal changes in 25(OH)D concentrations related to the season may have affected the results.

It is difficult to obtain enough vitamin D from the diet even if foods fortified with vitamin D are consumed.^[22]^ Therefore, adequate sunlight exposure is necessary to improve the vitamin D status. Godar et al^[23]^ investigated indoor-working adult Americans to calculate how much vitamin D3 they produced. They reported that certain adults with skin type II (22–59 years old) met their minimum vitamin D3 needs only during the summer with sun exposure over more than 30% of the body surface. Considering that skin type III is the predominant skin type in Korea,^[24]^ we presume that most indoor-working Korean adults do not produce sufficient amounts of vitamin D from sunlight exposure. Therefore, it is necessary to find an additional way to produce vitamin D.

Data regarding the efficiency of NBUVB-LED for producing 25(OH)D in healthy adults are lacking, although recent investigations have examined the possible role of NBUVB-LED exposure in increasing serum 25(OH)D levels. Vähävihu et al^[25]^ showed that 2 standard erythema doses of NBUVB exposure for 7 consecutive days improved vitamin balance in winter in healthy women with skin type II-III.^[26]^ Cicarma et al^[21]^ found that low-dose NBUVB treatment significantly increased vitamin D status. Karppinen et al^[27]^ showed that a suberythemal dose of NBUVB exposure to healthy subjects every second week over the winter months could maintain postsummer 25(OH)D concentrations. Based on these findings, we speculate that UVB therapy might influence the concentrations of 25(OH)D. Our study indicated that daily NBUVB-LED exposure could increase serum 25(OH)D concentrations, while exposure every other day was insufficient in affecting 25(OH)D levels. Whether this represents a true biological change, the effect of other factors, or simply increases in the mean is unknown. Thus, our results may partly support previous data and suggest that a larger study is needed.

This study had several limitations. First, the sample size was fairly small for valid statistical analysis to detect changes in 25(OH)D concentrations. In addition, 8 weeks were not long enough for the treatment response to reach a steady state. Second, NBUVB-LED exposure was conducted solely by education without any supervision by health personnel. Third, this study did not address UVB responses in obese participants. We already know that body fat decreases 25(OH)D in human skin. Thus, we would expect a lower 25(OH)D response to a given amount of UVB exposure in obese participants. However, confounding factors such as body fat mass were not well controlled. Fourth, there was no control with which to compare serum 25(OH)D levels. Fifth, the food intake of the subjects has not been fully assessed, which might affect serum 25(OH)D concentrations. However, we educated them to eat foods during the study as they usually had. Nevertheless, to the best of our knowledge, this was the first study to evaluate serum 25(OH)D concentrations using NBUVB-LED in Korea. A larger sample size is needed to examine the effect of NBUVB-LED on changes in serum 25(OH)D concentrations.

In conclusion, this pilot study indicated that NBUVB-LED exposure may increase serum 25(OH)D concentrations. Future studies should expand the number of participants and adjust for confounding factors. In addition, the safety of NBUVB-LED exposure should be assessed.

## Author contributions

All authors contributed to the data collection, interpretation, drafting, and editing of the manuscript.

## References

[1] Palacios C Gonzalez L. Is vitamin D deficiency a major global public health problem? J Steroid Biochem Mol Biol. 2014;144(Pt A):138–45. 24239505 10.1016/j.jsbmb.2013.11.003 PMC4018438

[2] De Martinis M Allegra A Sirufo MM. Vitamin D deficiency, osteoporosis and effect on autoimmune diseases and hematopoiesis: a review. Int J Mol Sci. 2021;22:8855. 34445560 10.3390/ijms22168855 PMC8396272

[3] Karadeniz Y Ozpamuk-Karadeniz F Ahbab S. Vitamin D deficiency is a potential risk for blood pressure elevation and the development of hypertension. Medicina (Kaunas). 2021;57:1297. 34946242 10.3390/medicina57121297 PMC8703486

[4] Yi Z Wang L Tu X. Effect of Vitamin D deficiency on liver cancer risk: a systematic review and meta-analysis. Asian Pac J Cancer Prev. 2021;22:991–7. 33906289 10.31557/APJCP.2021.22.4.991 PMC8325142

[5] Holick MF. Vitamin D deficiency. N Engl J Med. 2007;357:266–81. 17634462 10.1056/NEJMra070553

[6] Pearce SH Cheetham TD. Diagnosis and management of vitamin D deficiency. BMJ. 2010;340:b5664. 20064851 10.1136/bmj.b5664

[7] Nowson CA McGrath JJ Ebeling PR. Vitamin D and health in adults in Australia and New Zealand: a position statement. Med J Aust. 2012;196:686–7. 22708765 10.5694/mja11.10301

[8] Christakos S Dhawan P Verstuyf A. Vitamin D: metabolism, molecular mechanism of action, and pleiotropic effects. Physiol Rev. 2016;96:365–408. 26681795 10.1152/physrev.00014.2015 PMC4839493

[9] Wortsman J Matsuoka LY Chen TC. Decreased bioavailability of vitamin D in obesity. Am J Clin Nutr. 2000;72:690–3. 10966885 10.1093/ajcn/72.3.690

[10] Vernia F Valvano M Longo S. Vitamin D in inflammatory bowel diseases. Mechanisms of action and therapeutic implications. Nutrients. 2022;14:269. 35057450 10.3390/nu14020269 PMC8779654

[11] Vieth R. Why the optimal requirement for Vitamin D3 is probably much higher than what is officially recommended for adults. J Steroid Biochem Mol Biol. 2004;89–90:575–9. 10.1016/j.jsbmb.2004.03.038 15225842

[12] Balvers MG Brouwer-Brolsma EM Endenburg S. Recommended intakes of vitamin D to optimise health, associated circulating 25-hydroxyvitamin D concentrations, and dosing regimens to treat deficiency: workshop report and overview of current literature. J Nutr Sci. 2015;4:e23. 26090099 10.1017/jns.2015.10 PMC4463009

[13] Ashwell M Stone EM Stolte H. UK Food Standards Agency Workshop Report: an investigation of the relative contributions of diet and sunlight to vitamin D status. Br J Nutr. 2010;104:603–11. 20522274 10.1017/S0007114510002138

[14] Feldmeyer L Shojaati G Spanaus KS. Phototherapy with UVB narrowband, UVA/UVBnb, and UVA1 differentially impacts serum 25-hydroxyvitamin-D3. J Am Acad Dermatol. 2013;69:530–6. 23850091 10.1016/j.jaad.2013.04.058

[15] Norval M Bjorn LO de Gruijl FR. Is the action spectrum for the UV-induced production of previtamin D3 in human skin correct? Photochem Photobiol Sci. 2010;9:11–7. 20062839 10.1039/b9pp00012g

[16] Kalajian TA Aldoukhi A Veronikis AJ. Ultraviolet B light emitting diodes (LEDs) are more efficient and effective in producing vitamin D3 in human skin compared to natural sunlight. Sci Rep. 2017;7:11489. 28904394 10.1038/s41598-017-11362-2 PMC5597604

[17] Armas LA Dowell S Akhter M. Ultraviolet-B radiation increases serum 25-hydroxyvitamin D levels: the effect of UVB dose and skin color. J Am Acad Dermatol. 2007;57:588–93. 17637484 10.1016/j.jaad.2007.03.004

[18] Holick MF. Sunlight and vitamin D for bone health and prevention of autoimmune diseases, cancers, and cardiovascular disease. Am J Clin Nutr. 2004;80(Suppl 6):1678S–88S. 15585788 10.1093/ajcn/80.6.1678S

[19] van Weelden H De La Faille HB Young E. A new development in UVB phototherapy of psoriasis. Br J Dermatol. 1988;119:11–9. 3408653 10.1111/j.1365-2133.1988.tb07096.x

[20] Knaysi GA Crikelair GF Cosman B. The role of nines: its history and accuracy. Plast Reconstr Surg. 1968;41:560–3. 5654897

[21] Cicarma E Mork C Porojnicu AC. Influence of narrowband UVB phototherapy on vitamin D and folate status. Exp Dermatol. 2010;19:e67–72. 19849714 10.1111/j.1600-0625.2009.00987.x

[22] Wacker M Holick MF. Sunlight and vitamin D: a global perspective for health. Dermatoendocrinol. 2013;5:51–108. 24494042 10.4161/derm.24494 PMC3897598

[23] Godar DE Pope SJ Grant WB. Solar UV doses of adult Americans and vitamin D(3) production. Dermatoendocrinol. 2011;3:243–50. 22259652 10.4161/derm.3.4.15292 PMC3256341

[24] Youn JI CC Park SB Suh DH. The Fitzpatrick skin type in Korean people. Korean J Dermatol. 2000;920–7.

[25] Vahavihu K Ylianttila L Kautiainen H. Narrowband ultraviolet B course improves vitamin D balance in women in winter. Br J Dermatol. 2010;162:848–53. 20105173 10.1111/j.1365-2133.2010.09629.x

[26] Fitzpatrick TB. The validity and practicality of sun-reactive skin types I through VI. Arch Dermatol. 1988;124:869–71. 3377516 10.1001/archderm.124.6.869

[27] Karppinen T Ala-Houhala M Ylianttila L. Narrowband ultraviolet B exposures maintain vitamin D levels during winter: a randomized controlled trial. Acta Derm Venereol. 2016;96:490–3. 26524984 10.2340/00015555-2269

